# Lifetime ADHD symptoms highly prevalent in women with cardiovascular complaints. A cross-sectional study

**DOI:** 10.1007/s00737-023-01356-7

**Published:** 2023-08-18

**Authors:** L. S. ter Beek, M. N. Böhmer, M. E. Wittekoek, J. J. S. Kooij

**Affiliations:** 1 University Medical Center Utrecht, Utrecht, The Netherlands; 2 Dutch Expertise Center Adult ADHD at PsyQ, The Hague, The Netherlands; 3 HeartLife Clinics, Utrecht, The Netherlands

**Keywords:** ADHD, Women, Cardiovascular disease

## Abstract

Patients with Attention Deficit Hyperactivity Disorder (ADHD) are at greater cardiovascular risk. We investigated the association between ADHD symptoms and cardiovascular disease in women at a specialized Dutch cardiological clinic. Lifetime ADHD symptoms were found in 35% of women (*n* = 300) with cardiac complaints. Women with ADHD symptoms compared to those without were significantly younger but had no different cardiological profile. To protect women’s health, further research and multidisciplinary cooperation is required to better understand the relationship between ADHD and cardiovascular disease.

## Introduction

Cardiovascular disease (CVD) is the number one cause of death globally for women, accounting for more than 30% of all deaths in women (Z. Li et al. 2021). Both clinicians and patients still underestimate the cardiovascular risk in women, especially during perimenopause. Women with myocardial infarction may have an atypical presentation, that isthey are less likely to present with chest pain and more with pain between the shoulder blades, fatigue, and nausea (Vogel et al. 2021). Women are more susceptible to develop *diffuse* atherosclerotic disease, or Coronary Microvascular Dysfunction (CMD), which can lead to ischemia and myocardial infarction *without* obstructive coronary artery disease (Geraghty et al. 2021). Also several traditional cardiovascular risk factors such as smoking, hypertension, and diabetes mellitus have been associated with greater risk of CVD in women (Vogel et al. 2021). Risk factors unique to women, including gestational diabetes, polycystic ovarian syndrome, and premature ovarian failure are also associated with CVD. Unfortunately, female specific risk factors are currently not included in cardiovascular risk assessment tools.

Attention deficit hyperactivity disorder (ADHD) is a neurodevelopmental disorder characterized by a persistent, often lifetime pattern of inattention and/or impulsivity/hyperactivity that has a negative impact on daily functioning. The worldwide prevalence of ADHD is estimated to be around 5.9% in youth and 2.5% in adults (Faraone et al. 2021). Recent studies have shed light on the underrepresentation of females in clinical research on ADHD. Boys are three times more likely to receive an ADHD diagnosis than girls, while the ratio of adult males to females approaches 1.5:1.Girls with ADHD more often are diagnosed with the inattentive type of ADHD without showing disruptive behavior (Faheem et al. 2022). Research suggest that girls also may develop coping strategies and display socially adaptive behavior to mask their symptoms. Undetected girls and women yet are at higher risk for adverse outcomes, including impairments in physical and mental health, relationship difficulties and academic underachievement (Faheem et al. 2022).

Adult ADHD is accompanied by a broad range of both psychiatric comorbidities and physical conditions, such as sleep problems, mood disorders, substance abuse, obesity, diabetes, CVD, asthma, allergies, hypermobility, migraine, and dementia (Faraone et al. 2021). In addition, adults with ADHD more often have an unhealthy lifestyle such as smoking, alcohol, and drug use. A recent Swedish cohort study showed an increased risk for CVD in adults with ADHD of 38% versus 23% in people without ADHD (L. Li et al. 2022).

The associations found between CVD and ADHD in epidemiological research have never been studied in clinical practice in women with cardiovascular disease. We aimed to confirm this relationship at the Heartlife Clinics, a cardiological outpatient clinic in Utrecht, the Netherlands, that provides cardiovascular care focusing on both the prevention and treatment of heart disease with specialized expertise in women.. Given the underestimation of cardiovascular risk and underdiagnosis of ADHD in women, there were two main aims for the present study 1) to screen women with cardiovascular complaints on ADHD symptoms and 2) to assess whether ADHD symptoms in women is associated with an atypical cardiovascular profile.

## Methods

### Participants

In this retrospective cross-sectional study, clinical data was drawn from electronic health records of all female patients who applied to Heartlife Clinics between May 2021 and May 2022. Heartlife Clinics, a cardiological outpatient clinic in Utrecht, The Netherlands provides cardiovascular care focusing on both the prevention and treatment of heart disease with specialized expertise in women. It is estimated that women comprise 85% of the patients, with the largest age group between 40 and 60 years. Female patients aged 18 years and above with and without a history of cardiovascular disease, who were referred to Heartlife Clinics by their general practitioner or specialist were eligible for inclusion. Patients were asked to complete a baseline questionnaire including the Ultra-short questionnaire for ADHD prior to their first appointment.

### Measurements

#### Assessment of demographics and cardiovascular health indicators

Data collected from electronic health records included demographic information self-reported lifestyle related risk factors, history of diabetes, cardiac symptoms, physical examination, laboratory blood measurements, resting ECG, exercise stress test, carotid ultrasound measurements, and the diagnosis stable angina. Resting ECG was categorized as normal and abnormal. Examination of the carotid arteries was performed by an experienced cardiologist. The carotid ultrasound measurements were categorized as normal, abnormal carotid intima media thickening (thickened CIMT), and presence of one or more carotid plaques.

#### Assessment of ADHD

Patients were screened for lifetime ADHD symptoms using the Ultra-short questionnaire for ADHD (Ultra Korte Vragenlijst voor ADHD, UKV, Appendix)(Kooij 2022). It covers the frequent occurrence of the three core symptoms listed in DSM-5 for ADHD (Inattention, Impulsivity, and Hyperactivity) and one question about lifetime persistence of the symptoms. Each of the four items can be scored as 0 for “no” and 1 for “yes” with a maximum total score of 4. A score of 1 or more on the ADHD symptoms plus a score of 1 on the lifetime duration (chronicity) is indicative for one or more ADHD core symptoms, and thereby for one of the three subtypes of ADHD. UKV has been useful in clinical practice to identify patients with ADHD, but has not been validated in research.

## Results

This analysis included 300 female participants (age 56.90 ± 10.16 years) (Table 1). Based on the results of the UKV, a total of 105 35% of the women reported at least one chronic, lifetime ADHD core symptom (ADHD +), 21,7% reported two ADHD core symptoms and 9,3% reported three ADHD core symptoms to be chronically present from childhood.The ADHD + women were significantly younger than ADHD- women (55.29 years vs 57.77 years, *p* = 0.043). When corrected for the difference in age, ADHD + women were less likely to be a never drinker compared to ADHD- women (23% vs. 34%, *p* = 0.005). When corrected for age, fasting glucose levels were significantly lower in the ADHD + group compared with the ADHD-group (*p* = 0.033). None of the other outcomes showed significant differences between the ADHD + women and ADHD- women. ADHD symptoms were nosignificant predictor for atherosclerosis (*p* = 0.920) nor CMD (*p* = 0.403)(Table 2).

**Table 1:** Characteristics of the study population (*n* = 300)

	ADHD + *N* = 105	ADHD- *N* = 195	Total missing
Age in years, mean (SD)	55.29 (9.32)*	57.77 (10.50)	
BMI in kg/m^2^, mean (SD)	25.23 (3.91)	25.76 (4.37)	
Menopausal state, *N* (%)			6 (2)
Premenopausal	24 (23)	37 (19)	
Peri/postmenopausal	79 (77)	154 (81)	
Lifestyle, *N* (%)			
Smoking status			8 (2.7)
Current smoker	6 (6)	15 (8)	
Former smoker	42 (41)	55 (29)	
Never smoker	54 (53)	120 (63)	
Alcohol consumption			3 (1)
Current drinker	72 (69)	123 (64)	
Former drinker	8 (8)	4 (2)	
Never drinker	24 (23)**	66 (34)	
Drug usage			49 (16.3)
Current drug user	4 (5)	1 (.5)	
Former drug user	2 (2)	4 (2)	
Never user	77 (93)	163 (97)	
SBP in mm Hg, mean (SD)	125.96 (17.68)	128.50 (18.17)	
DBP in mm Hg, mean (SD)	81.57 (11.49)	82.51 (12.26)	
HR, mean (SD)	76.21 (12.99)	76.04 (14.80)	
Cardiac symptoms, *N* (%)			
Palpitations	51 (49)	86 (44)	1 (.3)
Chest pain	57 (54)	115 (59)	1 (.3)
Dyspnea	28 (27)	62 (32)	1 (.3)
Fatigue	31 (30)	56 (29)	2 (.7)
Diabetes mellitus, *N* (%)	3 (3)	5 (3)	
Total cholesterol in mmol/l, mean (SD)	5.62 (1.26)	5.68 (1.22)	41 (13.7)
LDL-c in mmol/l, mean (SD)	3.33 (1.07)	3.37 (1.05)	41 (13.7)
HDL-c in mmol/l, mean (SD)	1.70 (.45)	1.67 (.44)	42 (14)
Triglycerides, mean (SD))	1.37 (.84)	1.51 (.82)	47 (15.7)
TSH in mU/l, mean (SD)	1.51 (1.13)	1.76 (1.48)	169 (56.3)
T4 in pmol/l, mean (SD)	13.34 (1.41)	13.76 (1.92)	248 (82.7)
Non-fasting glucose in mmol/l, mean (SD)	5.53 (1.17)	5.56 (1.37)	247 (82.3)
Fasting glucose in mmol/l, mean (SD	4.92 (.24)*	5.59 (1)	257 (85.7)
ECG abnormalities, *N* (%)	35 (33)	85 (44)	1 (.3)
Exercise stress test, *N* (%)	70 (66.7)	109 (56.2)	20 (6.7)
Normal	27 (27)	49 (27)	
1. Hypertensive response	17 (17)	23 (13)	
2. Rhythm/conduction abnormalities	22 (22)	50 (27)	
3. Signs of ischemia	0	0	
4. Other ECG abnormalities	3 (3)	6 (3)	
Combination of 1 and 2	22 (22)	31 (17)	
Carotid ultrasound, *N* (%)			22 (7.3)
Normal	62 (64)	105 (58)	
Thickened CIMT	19 (20)	28 (15)	
Presence of plaques	16 (16)	48 (27)	
Stable angina, *N* (%)			
Functional (CMD)	23 (79)	41 (67)	
Obstructive	4 (14)	8 (13)	
Combination of the above	2 (7)	12 (20)	

*SD* = standard deviation, *BMI *= body mass index, *SBP *= systolic blood pressure, *DBP *= diastolic blood pressure, *HR *= heart rate, *LDL-c* = low density lipoprotein cholesterol, *HDL-c* = high density lipoprotein cholesterol, *TSH *= thyroid stimulating hormone, *T4 *= free thyroxine, *ECG* = electrocardiogram, *CIMT *= carotid intima media thickness, *CMD *= coronary microvascular dysfunction

Chi-square test for categorical variables and one-way ANOVA for continuous variables were performed

* < .05

** < .01

**Table 2:** Logistic regression for ADHD symptoms as predictor of atherosclerosis (*n* = 187) and CMD (*n* = 87)

Outcome		Model 1		Model 2	
		OR [95% CI]	*p*-value	OR [95% CI]	*p-*value
Atherosclerosis					
	ADHD	.816 [.437, 1.523]	.523	.944 [.473, 1.885]	.871
CMD					
	ADHD	1.610 [.558, 4.643]	.378	1.60 [.0532,4.815]	.403

OR = odds ratio, 95% CI = 95% confidence intervals. Artherosclerosis was defined as “yes” when thickened CMIT or presence of plaque op the carotid ultrasound, otherwise “no”. CMD (y/n) was defined as “yes” for Functional cause of stable angina, otherwise “no”

Model 1: unadjusted

Model 2: Model 1 plus adjusted for age and alcohol use

## Discussion

This is to our knowledge the first study investigating ADHD symptoms in women presenting with cardiovascular complaints in clinical cardiological practice. The main aim of this study was to screen for ADHD symptoms in women with cardiovascular complaints and to compare their cardiovascular profile with women without ADHD symptoms. Of the 300 women who were screened for ADHD symptoms, 105 (35%) screened positive, which is a 7 to tenfold increase compared to the prevalence of 3 to 5% in the general population. This high percentage of ADHD symptoms is adds to the previous findings of a prevalence of CVD of 38% in adults with ADHD in a recent population based Swedish Registry study (Du Rietz et al. 2021). In our study, the majority of the women were at peri-menopausal age, but the ADHD + women were significantly younger (-2 years) than ADHD- women, indicating earlier onset of cardiovascular complaints, pointing to increased severity in this group. Most women were not diagnosed with ADHD or aware of their ADHD symptoms, until the screening. The majority of women with stable angina suffered from functional or combined functional and obstructive cardiovascular disease and this did not differ between the two groups.

We theorize that the peri-menopause in women with ADHD symptoms plays a role in the onset of cardiac complaints. Previous research of our group suggests that women with ADHD are more likely to experience severe mood symptoms during peri-menopause than women without ADHD symptoms (Dorani et al. 2021). Declining levels of estrogen are associated with increased mood and ADHD symptoms, as well as with the onset of cardiovascular disease. Combined with lifestyle risk factors and exhaustion due to the chronic stress associated with ADHD and lifetime sleep problems, this clustering of events may have led to these adverse cardiovascular outcomes (van der Meer and Maas 2021). Further research is needed to confirm this.

Women may attribute their complaints of tiredness and exhaustion to chronic sleep loss that is associated with ADHD, and/or to peri-menopause, and overlook cardiac complaints, as they have learned to cope with their complaints and symptoms by continuing to go on, and not pay attention. Since adults with ADHD are at increased risk for several cardiovascular risk factors as well as anxiety and depression which induce psychological stress, we hypothesized that ADHD in women may contribute to increased rates of CMD (Coronary Microvascular Dysfunction) (functional stable angina). However, in this sample we cannot confirm this hypothesis.

## Strengths and limitations

To the best of our knowledge, this is the first study to examine the the prevalence of ADHD symptoms in women with cardiovascular health concerns in a clinical sample. In this study we did not include control women without cardiovascular problems, but we know the prevalence of ADHD is around 3–5% in the general population, in both males and females. Our study has some limitations. First, we included patients from a cardiac outpatient clinic with a special focus on women’s heart health rather than general cardiac care, therefore, in order to control for potential bias, screening for ADHD symptoms should be replicated in other cardiac centers Second, UKV was chosen because it is the shortest screener available, which is preferred by both patients and specialists in clinical practice, that measures the early onset and lifetime persistence of the three DSM-5 core symptoms of ADHD. Further, UKV is a screener but not a diagnostic tool, which may have overestimated the prevalence of ADHD symptoms in the present sample. Also, we were not able to fully correct for comorbidities (psychiatric disorders like depression), family history of CVD, female specific risk factors, and medication use that might influence the association between ADHD and CVD.

## Conclusion

Our results suggest added value of ADHD screening in women who are referred for cardiovascular examination. This high prevalence of ADHD symptoms in women at this cardiac clinic points to the need for more research, and alertness from both clinicians and the general public. Both CVD and ADHD symptoms in women are underdiagnosed and undertreated, and this combination is highly detrimental for the health outcomes of these women. It is crucial to improve the quality of life and achieve both prevention of cardiac risks, and optimal, combined cardiac and mental healthcare for women with ADHD, such as the Head, Heart and Hormones (H3) Network in the Netherlands.

## Appendix

**Ultrashort Questionnaire for ADHD in Adults (J.J.S. Kooij, 2006)**

1. **Do you usually feel restless?**

(e.g., nervous, difficulty sitting still, fidgeting, a lot of exercising, or being active) 

Yes/no

2. **Do you usually act first and then think?**

(e.g., blurting things out, spending too much money, or being impatient)Y

 Yes/no

3. **Do you usually have concentration problems?**

(e.g., being easily distracted, not finishing things, being easily bored, forgetful, or chaotic)

Yes/no

*If the answer to questions 1 and/or 2 and/or 3 is yes:*

4. **Have you always had this? (as long as you can remember, or have you been like this most of your life)**

Yes/no

*If the answer to question 4 is yes, please consider further diagnostic assessment for ADHD with the Diagnostic Interview for ADHD in adults (DIVA-5) (see* www.divacenter.eu *).*

Source: JJS Kooij. Adult ADHD. Diagnostic Assessment and Treatment, 4th ed. Springer, 2022.
